# An Emergency Medicine Disposition Challenge: A Scoping Review of Isolated Transverse Process Fractures of the Cervical Spine

**DOI:** 10.1016/j.acepjo.2026.100462

**Published:** 2026-07-13

**Authors:** Erin M. Harrington, Christopher S. Lozano, Alun D. Ackery, Christopher D. Witiw

**Affiliations:** 1 Temerty School of Medicine, University of Toronto, Ontario, Canada; 2 Division of Neurosurgery, St. Michael’s Hospital, University of Toronto, Toronto, Ontario, Canada; 3 Division of Emergency Medicine, St. Michael’s Hospital, University of Toronto, Toronto, Ontario, Canada

**Keywords:** blunt cerebrovascular injury, cervical spine trauma, transverse process fracture, vertebral artery

## Abstract

**Objectives:**

Isolated cervical transverse process fractures (TPFs) are increasingly identified in trauma. Although generally considered stable and nonoperative, current guidelines provide inconsistent recommendations on when to pursue spine consultation or computed tomography angiography (CTA) to assess for blunt cerebrovascular injury (BCVI). This scoping review aimed to determine which clinical or radiographic features of isolated cervical TPFs have been used or reported in association with spine consultation and/or BCVI screening.

**Methods:**

A comprehensive search of 5 databases and gray literature was conducted. Eligible studies included outcomes related to spine stability or BCVI. Data extraction focused on spine consultation, imaging, fracture characteristics, management, and BCVI related outcomes. Risk of bias was assessed using the ROBINS-E tool, and certainty of evidence was evaluated using the Grading of Recommendations Assessment, Development and Evaluation methodology. A narrative synthesis was performed due to heterogeneity.

**Results:**

Nine studies met inclusion criteria, comprising approximately 129 cases of isolated cervical TPFs. All fractures were managed nonoperatively, with variable collar usage. No studies explicitly reported rates of spinal instability or spine consultation. CTA use was variable, reflecting differences in imaging protocols. BCVI rates ranged from 0% to 23%, with multiple TPFs and fractures involving the transverse foramen (TF) being more frequent in cases positive for BCVI. Certainty of evidence for both outcomes was rated as very low due to methodological limitations and inconsistent reporting.

**Conclusion:**

Although there is insufficient evidence to make definitive recommendations on spine consultation or CTA for isolated cervical TPFs, available studies report exclusively nonoperative management without documented instability; however, the certainty of this evidence is very low. Multiple TPFs and fractures involving the TF were more frequently reported among BCVI cases, though findings were inconsistent. These findings suggest that emergency physicians should consider CTA in cases of multiple TPFs or TF involvement, while routine spine consultation for lesser injuries may be unnecessary; however, given the very low certainty of available evidence and medicolegal considerations surrounding spinal fractures, future prospective research is needed to support evidence-based guidelines and impact clinical practice.

## 1 Introduction

### 1.1 Background and Importance

Isolated cervical transverse process fractures (TPFs), once commonly missed on plain radiographs, are increasingly identified due to the improved sensitivity of computed tomography (CT).^1^ TPFs are common in trauma, particularly following high-energy mechanisms such as falls and motor vehicle collisions.^2^^,^^3^ Whole body CT has increased detection in polytrauma, further raising questions about their clinical significance and management in the emergency department (ED).^4^ Consequently, wider use of CT has increased detection of TPFs that may previously have gone undetected or remained clinically occult. Although many of these injuries may be stable or of limited direct clinical consequence, their potential association with more serious spinal, vascular, or ligamentous injury creates uncertainty around appropriate ED management.

TPFs that are truly isolated, meaning they do not extend into the lamina, pedicle, or facets, are considered stable, rarely causing neurologic deficits, and are managed conservatively.^2^^,^^5^ Yet, guidelines specific to these injuries are sparse and provide conflicting information. For example, the Western Trauma Association advises spine consultations for any cervical spine fracture.^6^ The British Orthopedic Association (BOA) attempts to be more specific by advising a consult for injuries that could cause spinal instability; however, they define any spine fracture as a risk for instability.^7^ Similarly, the American College of Surgeons (ACS) does not provide definitive guidance by stating that conservative management should be “considered” for “selected” patients with stable fractures, such as isolated TPFs.^8^ Consequently, spine consultations are still common in practice, reported in up to 72.9% of cases, despite a general belief that these fractures do not require surgical management.^9^ Furthermore, nuanced clinical scenarios, such as multiple or contiguous TPFs, which may reflect high-energy trauma and ligamentous injury, are not well addressed by current guidelines.^10^

Another concern is the association between cervical TPFs and BCVI, given their proximity to the vertebral artery. Guidelines conflict: ACS and BOA recommend screening all cervical fractures, while the Eastern Association for the Surgery of Trauma (EAST) recommends selective imaging limited to fractures involving C1 to C3 or the transverse foramen (TF), and conditional screening in lower-risk cases.^8^^,^^11^, ^12^, ^13^, ^14^ Both BOA and EAST base their guidance on the Denver criteria, which rely on very low-quality evidence and are evolving, initially recommending screening for all cervical fractures, later restricted to high-risk patterns, and more recently broadened again.^14^ This uncertainty leaves emergency physicians unsure when to order CT angiogram (CTA) in cases of isolated TPFs without other clear risk factors.^13^ The absence of clear guidelines for emergency physicians to use in managing these injuries likely drives variability in clinical practice and inconsistent thresholds for spine consultation and CT angiography across institutions, with potential for both over- and under-investigation.

### 1.2 Goals of This Investigation

Our review aims to investigate 2 key questions, what clinical or radiographic features of isolated cervical TPFs have been reported in association with (1) decisions to obtain spine specialist consultation and (2) decisions to perform CTA for BCVI.

## 2 Methods

This review was registered with PROSPERO on June 16^th^, 2025 (CRD420251075200). A search of 5 databases was conducted with institutional librarian support. The search terms included (isolated OR synonyms) AND (transverse process OR synonyms) AND (cervical OR synonyms) AND (fracture OR synonyms). Citation Chaser (Cardiff University, Cardiff, UK) was used to identify additional relevant studies and gray literature, including spine clearance guidelines and BCVI screening. All sources were last searched on June 26^th^, 2025. Although the study was initially designed as a systematic review, screening demonstrated significant heterogeneity in study populations, outcome definitions, and imaging protocols, as well as limited reporting of clinically relevant outcomes. Given these limitations, we determined that quantitative synthesis would not be appropriate and proceeded with a scoping review methodology focused on descriptive and narrative synthesis.

### 2.1 Eligibility Criteria

Included studies involved isolated cervical TPFs, which we defined as fractures limited to the transverse process(es) of a cervical vertebra(e), including fractures extending into the TF, without extension into the lamina, pedicle, or facets, and without other acute fractures of the cervical vertebra, and outcomes related to spinal instability or BCVI. Eligible studies either specifically examined isolated cervical TPFs or provided extractable data for a subgroup of patients with isolated fractures within a broader cohort. For studies that included mixed fracture populations, only data pertaining to patients meeting our definition of isolated TPFs were extracted. Screening questions were developed and pilot tested using a subset of records to ensure consistent interpretation. There were no exclusion criteria related to study date. All titles and abstracts were screened by one reviewer using intentionally broad inclusion criteria to minimize the risk of erroneous exclusion. Any record referring to cervical or spinal fractures was advanced to full-text review. Full-text reports for all potentially relevant studies were independently assessed by 2 reviewers. Discrepancies at both stages were resolved through discussion and, when necessary, adjudication by a third reviewer. Reviewers were not blinded to the study objectives. Formal inter-rater reliability statistics were not calculated. Where studies provided their own definition of “isolated,” these were extracted to capture variation across reports. Studies were grouped for synthesis based on their availability of data related to our 2 outcomes of interest.

### 2.2 Data Collection

Data extraction forms were developed and pilot tested. Data extraction was performed in Microsoft Excel by an independent reviewer pair, with discrepancies resolved by consensus or a third reviewer. Study characteristics, participant demographics, and clinical and fracture characteristics were collected. Regarding spine consultation, we extracted data on assessments and interventions related to spinal stability, such as rates of spine consultation/referral, dynamic or flexion–extension radiographs, dynamic instability, magnetic resonance imaging (MRI), discoligamentous injury, operative and nonoperative management, no treatment, conservative management failure, and discharge/admission. Regarding BCVI evaluation, we extracted rates of CTA, BCVI, fracture characteristics relevant to vascular risk, as well as BCVI management strategies, Biffl grade, and neurological event rates. No standardized criteria were applied for defining these aforementioned outcomes; data were extracted as reported by each study. All available reporting relevant to these outcome domains was included, regardless of definition or timeframe. Frequency data were extracted when provided.

### 2.3 Synthesis Methods

Risk of bias was assessed using the Risk Of Bias In Nonrandomized Studies - of Exposure (ROBINS-E) tool by one team member and verified by another.^15^ Certainty of evidence across studies for each key outcome was assessed using the Grading of Recommendations Assessment, Development and Evaluation (GRADE) working group methodology by 2 independent reviewers.^16^ Discrepancies at both stages were resolved through discussion and, when necessary, adjudication by a third reviewer. Reviewers were not blinded to the study objectives. Formal inter-rater reliability statistics were not calculated. A narrative synthesis grouped studies by reported outcomes: spine management, BCVI management, or both. We descriptively examined patterns in assessments, findings, and interventions, considering differences in populations, practices, and guidelines used. Sensitivity analyses were not conducted due to the descriptive nature of the synthesis and heterogeneous reporting.

## 3 Results

A total of 1123 results were identified through database searching, 519 additional records were identified through other sources, and 1140 records remained after duplicate deletion (Fig).^17^

**Figure:**
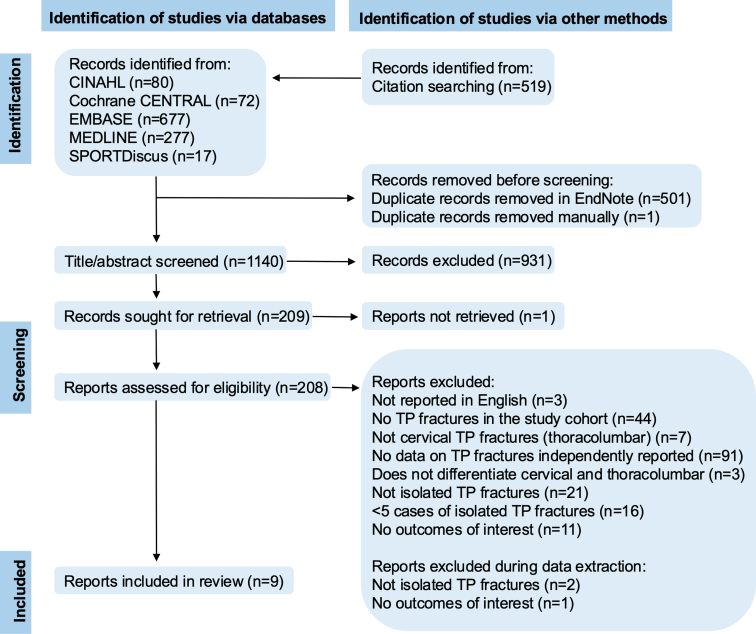
Preferred Reporting Items for Systematic reviews and Meta-Analyses (PRISMA) 2020 flow diagram.^17^

After screening, 9 articles were included, comprising approximately 129 cases of isolated cervical TPFs.^5^^,^^9^^,^^18^, ^19^, ^20^, ^21^, ^22^, ^23^, ^24^ These varied in their objectives, fracture descriptions, and reported outcomes, but were all based on trauma cohorts from Level 1 trauma centers (Table 1).^5^^,^^9^^,^^18^, ^19^, ^20^, ^21^, ^22^, ^23^, ^24^ Of the 9 included studies, only 3 studies provided independent demographic and presentation data (ie, age, sex, mechanism, symptoms, GCS), and just 1 presented Injury Severity Status (ISS) scores and associated injuries. No study reported data on ethnicity, comorbidities, or pre-existing spine conditions.

**Table 1:** Studies included in the scoping review of isolated TPFs of the cervical spine management.

Study	Overall risk of bias	Study design	“Isolated”definition	Sample size	Fracture characteristics described?	Spinal instability reported?	Spine consult reported?	BCVI reported?	CTA reported?
Bonney et al,^18^ 2017	Serious	Retrospective cohort study	“fractures limited in the cervical spine to the transverse process of one or multiple vertebrae”	45	Yes	No	No	Yes	Yes
Boulter et al,^19^ 2016	Moderate	Retrospective cohort study	Not explicitly defined	NR^a^	No	No	No	No	No
Bradley et al,^5^ 2008	Serious	Retrospective cohort study	“Isolated transverse process fractures were categorized by the presence of one or more transverse process fractures identified on CT scan. Transverse process fractures associated with other spinal injuries were characterized by the presence of one or more transverse process fractures in addition to any other acute fracture or dislocation in the cervical, thoracic, or lumbar spine”	5	No	No	No	Yes	Yes
Bui et al,^9^ 2017	Serious	Retrospective cohort study	"stable fractures that do not extend into the lamina, pedicle, or facet, Isolated transverse process fractures may be multilevel and/or associated with injuries outside of the spine"	9	No	No	No	Yes	No
Griffen et al,^20^ 2003	Low	Retrospective cohort study	Not explicitly defined	6	No	No	No	No	No
Khan et al,^21^ 2019	Moderate	Multi-site prospective cohort study	Not explicitly defined	5	Yes	No	No	No	No
Lebl et al, 2013^22^	Serious	Multi-site retrospective cohort study	Not explicitly defined	17	Yes	No	No	Yes	Yes
Oetgen et al,^23^ 2008^b^	Moderate	Retrospective cohort study	Not explicitly defined	21	Yes	No	No	Yes	Yes
Schotanus et al,^24^ 2010	Moderate	Prospective cohort study	“Isolated transverse process fractures of the subaxial cervical spine were defined as one or more transverse process fractures in the subaxial cervical spine without other affected structures in the subaxial cervical spine”	21	Yes	No	No	Yes	Yes

BCVI, blunt cerebrovascular injury; CT, computed tomography; CTA, computed tomography angiography; TF, transverse foramen; TPF, transverse process fracture.

a This study cohort of 306 participants, which included TPFs throughout the spine, did not explicitly specify the number of cervical TPFs within this cohort, but it reported no instances of operative treatment, which allowed us to extract this data point for the cervical subgroup.

b This study did not explicitly describe cases of TPFs but instead described isolated fractures involving the TF without subluxation or dislocation of the cervical spine, which is a subset of TPFs.

Risk of bias was rated as serious in 4 studies, moderate in 4, and low in 1 study using the ROBINS-E tool (Table 1,^5^^,^^9^^,^^18^, ^19^, ^20^, ^21^, ^22^, ^23^, ^24^ Table S1).^16^ Common concerns included lack of confounder adjustment (ie, ISS or pre-existing conditions that impact the spine or BCVI risk), missing data for excluded participants or those lacking follow-up, non-standardized outcome measurements, and possible selective reporting due to absence of pre-specified analysis plans.

### 3.1 Outcome #1: Spine Management

Six of the 9 included studies reported data relevant to spine management outcomes. None of these studies explicitly reported rates of spinal instability (Table 2).^5^^,^^9^^,^^19^, ^20^, ^21^^,^^24^

**Table 2:** Summary of surrogate outcome data related to spine management for isolated TPFs of the cervical spine.

Study	N (patients)	Operative management, n/N (%^a^)	Nonoperative management (collar), n/N (%^a^)	No treatment, n/N (%^a^)	Failure of conservative management, n/N (%^a^)
Boulter et al,^19^ 2016	NR^b^	(0)^b^	NR	NR	NR
Bradley et al,^5^ 2008	5	0/5 (0)	0/5 (0)	NR	NR
Bui et al,^9^ 2017	9	0/9 (0)	NR	NR	NR
Griffen et al,^20^ 2003	6	0/6 (0)	6/6 (100)	NR	NR
Khan et al,^21^ 2019	5	0/5 (0)	1/5 (20)	4/5 (80)	NR
Schotanus et al,^24^ 2010	21	0/21 (0)	0/21 (0)	NR	0/0 (0)

NR, not reported; TPF, transverse process fracture.

a Percentages (%) are of total number of patients unless otherwise specified.

b This study cohort of 306 participants, which included TPFs throughout the spine, did not explicitly specify the number of cervical TPFs within this cohort, but it reported no instances of operative treatment, which allowed us to extract this data point for the cervical subgroup.

No study systematically reported spine consultation rates. However, case-level details were available in 2 series. In Bui et al,^9^ all 4 patients with case-level data received consultation. Bradley et al^5^ described 5 cases, of which none required surgery, but 2 patients were initially placed in cervical collars and subsequently reviewed by the neurosurgical team, indicating specialist involvement.

No study explicitly reported the use of dynamic/flexion–extension radiographs or MRI. However, Bui et al described one patient who had associated interspinous and nuchal ligament injuries, managed with a collar and physical therapy. Bradley et al observed that upright radiographs were often obtained when other acute spine injuries were present but rarely performed in the setting of isolated TPFs; however, they did not quantify this.

All studies described exclusively nonoperative management. Collar use was reported in 4 studies, with rates ranging from 0% to 100%. Bui et al reported collar use in all 4 patients with follow-up, including 1 who also underwent physical therapy. Khan et al^21^ reported a high rate of no intervention (4/5 patients, 80%), while all patients described by Schotanus et al^24^ were permitted unrestricted movement. No study reported admission or ED discharge outcomes.

Only Schotanus et al explicitly reported failure rates of conservative management, which were 0% (0/21 patients). Applying the rule of 3 for zero-event studies, the upper bound of the approximate 95% confidence interval would be 14%.^25^ Long-term outcomes were available in 1 study, which included 59 patients with isolated TPFs across the spine (cervical and thoracolumbar).^19^ Of the patients with data at last follow-up, all patients were neurologically intact, and 97.8% were ambulatory. The mean follow-up duration was 5.5 months, and 37.3% of patients had no follow-up. These outcomes were not stratified by cervical versus thoracolumbar injuries.

### 3.2 Outcome #2: BCVI management

Six studies explicitly reported rates of CTA usage and/or BCVI incidence (Table 3).^5^^,^^9^^,^^18^^,^^22^, ^23^, ^24^ In the samples we extracted from Lebl et al^22^ and Oetgen et al^23^ all cases involved either multiple TPFs or fractures involving the TF, respectively.^,^

**Table 3:** Summary of key and surrogate outcome data related to BCVI management for isolated TPFs of the cervical spine.

Study	N (patients)	CTA performed? n/N (%^a^)	BCVI?, n/N (%^a^)	at least one TPF involving the TF? n/N (%^a^)	Fracture comminution, n/N (%^a^)	Intraforaminal fragment, n/N (%^a^)	Neurological event secondary to BCVI? n/N (%^a^)
Bonney et al,^18^ 2017	45	45/45 (100)	5/45 (11.1)	23/45 (51.1)	NR	NR	NR
Bradley et al,^5^ 2008	5	2/5 (40)	0/5 (0)	NR	NR	NR	NR
Bui et al,^9^ 2017	9	NR	1/9 (11.1)	NR	NR	NR	NR
Lebl et al,^22^ 2013	17	17/17 (100)	4/17 (23.5)	NR	NR	NR	0/17 (0)
Oetgen et al,^23^ 2008^b^	21	21/21 (100)	5/21 (23.8)	21/21 (100)	5/21 (23.8)	4/21 (19.0)	NR
Schotanus et al,^24^ 2010	21	0/21 (0)	NR	0	NR	NR	NR

BCVI, blunt cerebrovascular injury; CTA, computed tomography angiography; NR, not reported; TF, transverse foramen; TPF, transverse process fracture.

a Percentages (%) are of total number of patients unless otherwise specified.

b This study did not explicitly describe cases of TPFs but instead described isolated fractures involving the TF without subluxation or dislocation of the cervical spine, which is a subset of TPFs.

Five studies reported CTA use: 3 reported a 100% usage rate (45/45, 17/17, and 21/21 patients) (in cohorts consisting only of patients who underwent BCVI screening or at a site that routinely orders CTA for all cervical spine fractures), one had a 40% rate (2/5 patients), and one had 0% CTA rate (0/21 patients).^5^^,^^18^^,^^22^, ^23^, ^24^ The latter study explained that no fractures involved the TF, so vertebral artery injury was not considered a risk.^24^

Five studies reported BCVI incidence: 2 reported rates of ∼11% (5/45 and 1/9 patients), 2—of∼23% (4/17 and 5/21 patients), and 1 study found no instances of BCVI with isolated TPFs (0/5 patients) (nor any instances of BCVI with other fracture types either).^5^ Although not explicitly reported on by Schotanus et al, their study included detailed follow-up data from hospital admissions and stated that there were no clinical signs of vertebral artery injury in their sample. Bui et al reported one asymptomatic case of BCVI (1/9 TPF cases). Given the small sample, the approximate upper bound of the 95% confidence interval using a rule-of-3 approach is 44% (∼4/9), indicating substantial imprecision. Lebl et al reported that 23% of their cohort (4/17 patients) had a BCVI, but 0% had neurologic sequelae during hospitalization. However, given the small number of affected patients, the approximate upper 95% confidence bound for neurologic sequelae is 75% (∼3/4), reflecting substantial imprecision.

Of the studies reporting BCVI related outcomes, only 4 described fracture characteristics. Three provided separate data for TPFs associated with BCVI, but the only consistently reported variable was multiple TPFs (reported as multilevel fractures in the study by Oetgen et al) (Table 4^).18,22,23^ These were common in BCVI cases (80%–100%), but less frequent in non-BCVI cases (∼20%), except in the study by Lebl et al, in which all cases (BCVI and non-BCVI) had multiple TPFs.^22^

**Table 4:** Characteristics of isolated cervical TPFs associated with BCVI.

	Bonney et al,^18^ 2017		Lebl et al,^22^ 2013		Oetgen et al,^23^ 2008^a^	
	BCVI -	BCVI +	BCVI -	BCVI +	BCVI -	BCVI +
Patients						
N (patients)	40	5	13	4	16	5
Age, y (mean)	46.2	48.8	NR	NR	NR	NR
Age (SD)	16.4	27.2	NR	NR	NR	NR
Age (range)	19-74	21-88	NR	NR	NR	NR
Male, n/N (%^b^)	NR	4/5 (80)	NR	NR	NR	NR
Female, n/N (%^b^)	NR	1/5 (20)	NR	NR	NR	NR
Fracture characteristics						
Fracture displacement (mm) (mean)	NR	NR	NR	NR	1.63	2.59
Fracture displacement (mm) (range)	NR	NR	NR	NR	1.13-2.13	1.69-3.49
Fracture comminution, n/N (%^b^)	NR	NR	NR	NR	2/16 (12.5)	3/5 (60)
Intraforaminal fragment, n/N (%^b^)	NR	NR	NR	NR	3/16 (18.8)	1/5 (20)
No. of TPFs						
No. (mean)	1.4	3.0	NR	NR	NR	NR
No. (range)	1-4	2-4	NR	NR	NR	NR
1 fracture, n/N (%^b^)	30/40 (75)	0/5 (0)	NR	NR	NR	NR
>1 fracture, n/N (%^b^)	10/40 (25)	5/5 (100)	NR	NR	NR	NR
Multiples						
Multiple TPFs, n/N (%^b^)	10/40 (25)	5/5 (100)	13/13 (100)	4/4 (100)	NR	NR
Bilateral TPFs, n/N (%^b^)	NR	2/5 (40)	NR	NR	NR	NR
Multilevel TPFs, n/N (%^b^)	NR	5/5 (100)	NR	NR	3/16 (18.8)	4/5 (80)
Contiguous multiples, n/N (%^b^)	NR	5/5 (100)	NR	NR	NR	NR
TF involvement						
No. involving the TF (mean)	0.63	2.0	NR	NR	NR	NR
No. of fractures into the TF (range)	0-4	0-4	NR	NR	NR	NR
0 fractures into the TF, n/N (%^b^)	21/40 (52.5)	1/5 (20)	NR	NR	0/16 (0)	0/0 (0)
1+ fractures into the TF, n/N (%^b^)	19/40 (47.5)	4/5 (80)	NR	NR	16/16 (100)	5/5 (100)
Vertebral level						
High cervical levels, n/N (C1-C3) (%^b^)	NR	0/0 (0)	NR	NR	NR	NR
C4, n/N (%^b^)	NR	1/5 (20)	NR	NR	NR	NR
C5, n/N (%^b^)	NR	4/5 (80)	NR	NR	NR	NR
C6, n/N (%^b^)	NR	4/5 (80)	NR	NR	NR	NR
C7, n/N (%^b^)	NR	4/5 (80)	NR	NR	NR	NR

BCVI, blunt cerebrovascular injury; TF, transverse foramen; TPF, transverse process fracture.

a This study did not explicitly describe cases of TPFs but instead described isolated fractures involving the TF without subluxation or dislocation of the cervical spine, which is a subset of TPFs.

b Percentages (%) are of total number of patients in the BCVI− or BCVI + group.

Bonney et al^18^ found significant differences between BCVI and non-BCVI cases for number of fractures, presence of a single fracture, and number of fractures involving the TF, with number of fractures being the strongest predictor. In contrast, Lebl et al found multiple TPFs were not an independent predictor of BCVI. Oetgen et al, whose sample included only TPFs involving the TF, found significant associations between BCVI and multilevel fractures (OR 17.3, 95% CI 1.39-216.61, *P* =.027) and fracture comminution (OR 10.5, 95% CI 1.03-107.17, *P* = .047) but not displacement, or intraforaminal fragments (*P* > .05). However, as odds ratios are not ideal for cohort data, these should not be interpreted as definitive measures of risk. Notably, unadjusted risk ratios calculated from raw data are smaller than reported odds ratios (RR 8.0 and RR 4.8 for multilevel fractures and fracture comminution, respectively).

All included studies were observational, resulting in the certainty of the evidence for each outcome starting at low (Table 5).^15^^,^^16^ For our first question surrounding spine consultation, the certainty was downgraded to very low, due to moderate-to-serious risk of bias in most studies from inadequate confounder adjustment, incomplete outcome reporting, non-standardized assessments, indirectness from differences in study populations and definitions of instability compared with our review objectives, imprecision due to small sample sizes, and variable or absent outcome reporting. For the question surrounding BCVI, the certainty of evidence was also downgraded to very low due to serious risk of bias in most studies from imprecision due to small sample sizes and wide confidence intervals, and indirectness from differences in study populations and BCVI definitions.

**Table 5:** GRADE evidence profile table for each outcome.

Outcome	No. of studies	Risk of bias	Inconsistency	Indirectness	Imprecision	Publication bias	Certainty
Spine management	6	Low-Serious	No serious inconsistency	Serious indirectness	Very serious imprecision	No serious risk	Very low
BCVI management	6	Moderate-Serious	No serious inconsistency	Serious indirectness	Serious imprecision	No serious risk	Very low

BCVI, blunt cerebrovascular injury; GRADE, Grading of Recommendations Assessment Development and Evaluation.

## 4 Limitations

The main limitation was the inability to conduct meta-analysis due to reporting heterogeneity. Although 4 studies reported both CTA use and BCVI detection, differences in imaging criteria limited comparability. In Lebl et al, Oetgen et al, and Bonney et al only patients who underwent CTA were included, with clinician discretion guiding screening decisions in the former 2 and a universal CTA policy guiding decisions in the latter. Bradley et al reported 40% CTA use but did not define decision criteria. These differences suggest varying pretest probabilities. For the outcome of spine management, we descriptively extracted data on spine service involvement, additional imaging, and treatment approaches as surrogate markers of management patterns. We acknowledge that these variables may reflect institutional protocols, provider practice variation, or resource availability rather than true clinical concern for instability. We also acknowledge that these variables are imperfect proxies for spine consultation or concern, as decision making could not be reliably attributed to specialist involvement versus emergency physician management, limiting any ability to draw conclusions regarding consultation practices. The decision to conduct a scoping review rather than a full systematic review with meta-analysis reflects the substantial heterogeneity and limited methodological rigor of available studies, which precluded meaningful quantitative synthesis.

Other limitations include the small sample size, which limits power to detect rare but meaningful outcomes; the retrospective, single-center design of most studies, raising concerns of selection bias, incomplete data capture, and generalizability; inconsistent or absent definitions of “isolated”, and our lack of formal inter-rater reliability for screening and data extraction. Important patient-level variables were often missing, constraining confounding assessments, and no studies addressed patient-reported outcomes. Selective reporting is also possible, as TPFs embedded within larger trauma cohorts may be more likely to be published when associated with complications. Additionally, isolated findings reported in single cases, such as the single case of ligamentous injury reported by Bui et al should be interpreted cautiously, as individual case-level observations cannot establish prevalence, causal association, or clinical significance. Furthermore, most included studies had unclear patient follow-up, which severely limits any conclusions regarding the long-term safety of the management strategies.

## 5 Discussion

### 5.1 Interpretation of Findings

This review addressed 2 key questions: how isolated cervical TPFs have been evaluated with respect to spine consultation, and how CTA has been applied to screen for BCVI. Only one prior systematic analysis addressed these fractures, mainly supporting nonoperative management.^2^ Our review extends these findings by including 7 additional studies examining isolated cervical TPFs, more detailed characterization of fracture patterns, as well as the important outcome of BCVI, which was not included in this prior review. Across our 9 studies (approximately 129 cases), all TPFs were managed nonoperatively, with varied immobilization and CTA use. Multiple fractures were more frequent in BCVI cases, though evidence certainty was very low.

### 5.2 Comparison with Previous Studies

In our review, only 3 studies specifically sought out to address spine management and stability. Schotanus et al concluded that these fractures are stable, requiring neither surgery nor routine immobilization. Boulter et al^19^ supported conservative management without mandatory consultation, and Bradley et al likewise found no evidence of instability or operative need. Although consultation rates and use of surrogate markers (ie, MRI and dynamic radiographs) were not explicitly reported, rare discoligamentous injuries underscore the importance of identifying red-flag features. The consistent absence of adverse outcomes in this review supports the safety of nonoperative management; however, given very low-certainty evidence, no strong recommendation can be made for or against routine spine consultation, as consultation practices were inconsistently reported and may occur even when management is nonoperative.

Our review highlights BCVI as a valid clinical concern. The wide range in reported BCVI incidence likely reflects heterogeneity in inclusion criteria, definitions of BCVI, imaging protocols, and potential workup bias, related to variable CTA screening thresholds across institutions. For example, studies that screened all TPFs likely captured lower-risk cohorts than those that only imaged patients with TF involvement or symptoms. This variability underscores the difficulty of estimating true incidence and highlights the need for standardized definitions and universal screening strategies in future research. Importantly, in patients without TF involvement or multilevel TPFs, reported BCVI rates were lower, but small numbers and heterogeneous reporting limit precision and preclude definitive conclusions. Given very low-certainty evidence, we cannot strongly recommend for or against routine CTA. Screening is likely reasonable, particularly when the fracture involves the TF (per Denver and EAST criteria), or when multiple TPFs are present, which is highlighted as a potential BCVI risk factor in this review.^13^^,^^14^

### 5.3 Clinical Implications

This review highlights the lack of targeted research on these injuries. Most existing evidence is embedded within studies of mixed fracture patterns or broader spinal injury cohorts. This matters, as current guidelines and practices vary widely, creating potential for both overzealous management and inefficient health care resource use. Notably, some centers report routinely performing CTA for all cervical spine fractures, whereas others employ selective screening strategies, suggesting that universal screening may be overinclusive for truly isolated TPFs without transverse foraminal involvement or other risk factors. This inconsistency may reflect differing guideline scope and specificity, and the lack of fracture-specific guidance for isolated TPFs. Furthermore, although existing screening tools such as NEXUS and the Canadian C-spine Rule remain foundational decision support tools for cervical spine clearance, their definitions of clinically important injury were developed before the widespread use of CT as the dominant imaging modality for cervical spine trauma.^26^^,^^27^ The convention that isolated TPFs are clinically insignificant largely reflects earlier outcome definitions focused on injuries requiring stabilization or specialized follow-up.^28^ Although this framework supports the generally stable nature of most isolated TPFs, it may not fully address contemporary questions regarding uncommon associated findings such as transverse foraminal involvement, multilevel TPFs, BCVI, or occult ligamentous injury. This distinction may help explain why uncertainty persists regarding ED management despite prior classification of isolated TPFs as clinically insignificant.

These findings may also have implications for transfer and triage decisions. In the absence of neurologic deficit, transverse foraminal involvement, multilevel injury, or other concerning associated findings, isolated cervical TPFs alone may not warrant transfer solely for spine specialist evaluation; however, the limited evidence base prevents definitive recommendations, and local resources, imaging availability, and trauma system protocols should guide disposition.

These limitations demonstrate the need for future research to better define true BCVI risk and inform standardized imaging protocols. Routine spine consultation or immobilization may be unnecessary for many, diverting resources from higher-risk patients. However, this conclusion is uncertain due to the limited and heterogeneous evidence. Conversely, the true BCVI risk in this subgroup remains uncertain. A “shotgun” approach of performing CTA in all patients may detect rare injuries but exposes many low-risk patients to radiation and contrast, while imposing additional health care costs. Balancing low absolute risk with the severe consequences of missed BCVI remains a central clinical challenge.

Most existing evidence comes from broader trauma cohorts not designed to address isolated cervical TPFs, and as such, the true risk of spinal instability remains uncertain. Although all reported fractures in our review were managed nonoperatively without adverse outcomes, the small sample sizes, inconsistent reporting, and heterogeneous protocols prevent strong conclusions about the necessity of routine spine consultation or immobilization. Similarly, the use of CTA for BCVI carries potential harms, including radiation exposure and contrast-induced nephropathy, as well as additional health care costs; although the absolute risk is generally low, it is not negligible. Decisions regarding consultation or imaging must therefore balance the limited evidence of clinical benefit against these potential risks and costs, emphasizing the need for individualized clinical judgment and further research. As such, the primary contribution of this review is to highlight the limitations of the current evidence rather than to support definitive changes in clinical practice.

### 5.4 Research Implications

This review identified the need for targeted, high-quality studies to generate consistent, evidence-based guidelines that optimize patient safety while avoiding unnecessary interventions. Most existing evidence came from broader trauma cohorts not designed to address our specific research questions. As such, critical patient and presentation characteristics that influence spinal instability or BCVI risk were often missing. Notably, impaired neurological status plays a role in BCVI risk stratification and may influence spine consultation decisions in unexaminable patients.^6^^,^^14^ These missing data limit the ability to fully assess risk and reinforce the need for well-designed multicenter studies, ideally prospective, to capture comprehensive patient characteristics and employ universal screening protocols to better estimate true BCVI incidence. Capturing this data could provide the evidence necessary to inform guidelines and optimize management strategies for isolated cervical TPFs.

In conclusion, there is insufficient evidence to make definitive recommendations on spine consultation or CTA for isolated cervical TPFs, nor is there enough evidence to reliably quantify the risks associated with these injuries. Although nonoperative management appears safe, inconsistent reporting and imaging protocols contribute to ongoing clinical uncertainty. Consequently, emergency physicians are forced to make decisions amid limited evidence. Future research is needed to support clearer, evidence-based guidelines.

## Author Contributions

ADA and CDW had the idea for the article. EMH performed the literature search. EMH and CSL performed article screening, data extraction, and data analysis. EMH drafted the work, and all authors critically revised the work. All co-authors have had the opportunity to review the final manuscript and have provided their permission to publish the manuscript.

## Funding and Support

By *JACEP Open* policy, all authors are required to disclose any and all commercial, financial, and other relationships in any way related to the subject of this article as per ICMJE conflict of interest guidelines (see www.icmje.org). This study did not receive any funding.

## Conflict of Interest

All authors have affirmed they have no conflicts of interest to declare.

## References

[1] The Trauma Quality Programs Best Practices Project Team The ACS TQP best practices guidelines. Cervical spine imaging. https://www.facs.org/media/oxdjw5zj/imaging_guidelines.pdf.

[2] Nagasawa D.T., Bui T.T., Lagman C. (2017). Isolated transverse process fractures: a systematic analysis. World Neurosurg.

[3] Jessula S., Yanchar N.L., Romao R., Green R., Asbridge M. (2022). Where to start? Injury prevention priority scores for traumatic injuries in Canada. Can J Surg.

[4] Long B., April M.D., Summers S.M., Koyfman A. (2017). Whole body CT versus selective radiological imaging strategy in trauma: an evidence-based clinical review. Am J Emerg Med.

[5] Bradley L.H., Paullus W.C., Howe J., Litofsky N.S. (2008). Isolated transverse process fractures: spine service management not needed. J Trauma.

[6] Ciesla D.J., Shatz D.V., Moore E.E. (2020). Western Trauma Association critical decisions in trauma: cervical spine clearance in trauma patients. J Trauma Acute Care Surg.

[7] British Orthopedic Association British orthopedic association standards for trauma and orthopedics. https://www.boa.ac.uk/static/1e3aff2a-576f-484b-ab21ec91300678cf/BOAST-Cervical-Spine-Clearance.pdf.

[8] The Trauma Quality Programs Best Practices Project Team The ACS TQP best practices guidelines. Nonoperative management. https://www.facs.org/media/k45gikqv/spine_injury_guidelines.pdf.

[9] Bui T.T., Nagasawa D.T., Lagman C. (2017). Isolated transverse process fractures and markers of associated injuries: the experience at University of California, Los Angeles. World Neurosurg.

[10] Alijanipour P., Greif D., Lebwohl N.H., Gjolaj J.P. (2020). Isolated multiple lumbar transverse process fractures with spinal instability: an uncommon yet serious association. Eur Spine J.

[11] British Orthopedic Association British orthopedic association standards for trauma and orthopedics. https://www.boa.ac.uk/standards-guidance/boasts.html.

[12] Cothren C.C., Moore E.E., Ray C.E., Johnson J.L., Moore J.B., Burch J.M. (2007). Cervical spine fracture patterns mandating screening to rule out blunt cerebrovascular injury. Surgery.

[13] Kim D.Y., Biffl W.L., Bokhari F. (2020). Evaluation and management of blunt cerebrovascular injury: a practice management guideline from the Eastern Association for the Surgery of Trauma. J Trauma Acute Care Surg.

[14] Geddes A.E., Burlew C.C., Wagenaar A.E. (2016). Expanded screening criteria for blunt cerebrovascular injury: a bigger impact than anticipated. Am J Surg.

[15] Higgins J.P.T., Morgan R.L., Rooney A.A. (2024). A tool to assess risk of bias in non-randomized follow-up studies of exposure effects (ROBINS-E). Environ Int.

[16] Bezerra C.T., Grande A.J., Galvão V.K., Santos D.H.M.D., Atallah Á.N., Silva V. (2022). Assessment of the strength of recommendation and quality of evidence: GRADE checklist. A descriptive study. Sao Paulo Med J.

[17] Page M.J., McKenzie J.E., Bossuyt P.M. (2021). The PRISMA 2020 statement: an updated guideline for reporting systematic reviews. BMJ.

[18] Bonney P.A., Burks J.D., Conner A.K. (2017). Vertebral artery injury in patients with isolated transverse process fractures. J Clin Neurosci.

[19] Boulter J.H., Lovasik B.P., Baum G.R. (2016). Implications of isolated transverse process fractures: is spine service consultation necessary?. World Neurosurg.

[20] Griffen M.M., Frykberg E.R., Kerwin A.J. (2003). Radiographic clearance of blunt cervical spine injury: plain radiograph or computed tomography scan?. J Trauma.

[21] Khan A.D., Liebscher S.C., Reiser H.C. (2019). Clearing the cervical spine in patients with distracting injuries: an AAST multi-institutional trial. J Trauma Acute Care Surg.

[22] Lebl D.R., Bono C.M., Velmahos G.C., Metkar U., Nguyen J.T., Harris M.B. (2013). Vertebral artery injury associated with blunt cervical spine trauma: a multivariate regression analysis. Spine.

[23] Oetgen M.E., Lawrence B.D., Yue J.J. (2008). Does the morphology of foramen transversarium fractures predict vertebral artery injuries. Spine (Spila Pa 1976).

[24] Schotanus M., Van Middendorp J.J., Hosman A.J.F. (2010). Isolated transverse process fractures of the subaxial cervical spine: a clinically insignificant injury or not?: a prospective, longitudinal analysis in a consecutive high-energy blunt trauma population. Spine (Spila Pa 1976).

[25] Hanley J.A., Lippman-Hand A. (1983). If nothing goes wrong, is everything all right? Interpreting zero numerators. JAMA.

[26] Hoffman J.R., Mower W.R., Wolfson A.B., Todd K.H., Zucker M.I. (2000). Validity of a set of clinical criteria to rule out injury to the cervical spine in patients with blunt trauma. National Emergency X-Radiography Utilization Study Group. N Engl J Med.

[27] Mower W.R., Wolfson A.B., Hoffman J.R., Todd K.H. (2004). The Canadian C-spine rule. N Engl J Med.

[28] Stiell I.G., Lesiuk H.J., Vandemheen K.L. (1999). Obtaining consensus for a definition of "clinically important cervical spine injury" in the CCC study. Acad Emerg Med.

