# Supplementary material for: An Emergency Medicine Disposition Challenge: A Scoping Review of Isolated Transverse Process Fractures of the Cervical Spine
Source: J Am Coll Emerg Physicians Open. 2026 Jul 13;7(5):100462. doi: 10.1016/j.acepjo.2026.100462 (PMC13382314; doi:10.1016/j.acepjo.2026.100462)
Supplement: Supplementary Table 1 [file mmc1.docx]

**SUPPLEMENTARY MATERIALS**

**Table S1.** The Risk Of Bias In Non-randomized Studies - of Exposure (ROBINS-E) tool assessments for included studies.

| **Domains** | **Bonney et al., 2017** | **Boulter et al., 2016** | **Bradley et al., 2008** | **Bui et al., 2017** | **Griffen et al., 2003** | **Khan et al., 2019** | **Lebl et al., 2013** | **Oetgen et al., 2008** | **Schotanus et al., 2010** |
| --- | --- | --- | --- | --- | --- | --- | --- | --- | --- |
| Confounding | Serious | Moderate | Serious | Serious | Low | Low | Moderate | Moderate | Moderate |
| Measurement of the exposure | Low | Low | Low | Low | Low | Low | Low | Low | Low |
| Selection of participants | Moderate | Low | Low | Low | Low | Low | Serious | Moderate | Low |
| Post-exposure interventions | Low | Low | Low | Low | Low | Low | Low | Low | Low |
| Missing data | Moderate | Moderate | Moderate | Serious | Low | Low | Low | Moderate | Moderate |
| Measurement of the outcome | Low | Moderate | Moderate | Moderate | Low | Low | Low | Low | Low |
| Selection of the reported result | Moderate | Low | Moderate | Moderate | Low | Moderate | Moderate | Low | Low |
| Overall risk of bias | Serious | Moderate | Serious | Serious | Low | Moderate | Serious | Moderate | Moderate |
